# Development of a validated algorithm for the diagnosis of paediatric asthma in electronic medical records

**DOI:** 10.1038/npjpcrm.2016.85

**Published:** 2016-11-24

**Authors:** Andrew J Cave, Christina Davey, Elaheh Ahmadi, Neil Drummond, Sonia Fuentes, Seyyed Mohammad Reza Kazemi-Bajestani, Heather Sharpe, Matt Taylor

**Affiliations:** 1 Department of Family Medicine, University of Alberta, Edmonton, AB, Canada

## Abstract

An accurate estimation of the prevalence of paediatric asthma in Alberta and elsewhere is hampered by uncertainty regarding disease definition and diagnosis. Electronic medical records (EMRs) provide a rich source of clinical data from primary-care practices that can be used in better understanding the occurrence of the disease. The Canadian Primary Care Sentinel Surveillance Network (CPCSSN) database includes cleaned data extracted from the EMRs of primary-care practitioners. The purpose of the study was to develop and validate a case definition of asthma in children 1–17 who consult family physicians, in order to provide primary-care estimates of childhood asthma in Alberta as accurately as possible. The validation involved the comparison of the application of a theoretical algorithm (to identify patients with asthma) to a physician review of records included in the CPCSSN database (to confirm an accurate diagnosis). The comparison yielded 87.4% sensitivity, 98.6% specificity and a positive and negative predictive value of 91.2% and 97.9%, respectively, in the age group 1–17 years. The algorithm was also run for ages 3–17 and 6–17 years, and was found to have comparable statistical values. Overall, the case definition and algorithm yielded strong sensitivity and specificity metrics and was found valid for use in research in CPCSSN primary-care practices. The use of the validated asthma algorithm may improve insight into the prevalence, diagnosis, and management of paediatric asthma in Alberta and Canada.

## Introduction

An understanding of the prevalence and management of paediatric asthma is hindered by issues with disease definition and diagnosis. As there is no standard definition of the type, severity, or frequency of symptoms, the diagnosis of asthma in young children is challenging.^1^ Lung function measurements to assess airflow limitation or airway inflammation are also unreliable in young children under age six.^1,2^ Family physicians’ electronic medical records (EMRs) provide a rich source of clinical data that can be used in chronic disease surveillance and in determining the effectiveness of disease prevention and management interventions. However, the use of EMR data to identify paediatric patients with asthma cannot be successful without first confirming that a definition and case-finding diagnostic algorithm is valid.

The Canadian Primary Care Sentinel Surveillance Network (CPCSSN) has developed a process that enables data from 12 different EMR databases to be extracted, cleaned and merged into a single primary-care data set.^3^ The use of this data is intended to enhance patient care by improving understandings of the epidemiology of selected chronic illnesses.^3^ To date, case definitions have been developed and validated for eight common chronic conditions, including diabetes, chronic obstructive pulmonary disease (COPD) and dementia.^4^ The purpose of this study was to develop and validate a case definition and case-finding algorithm to identify children with asthma who consult family physicians, in order to provide accurate estimates of childhood asthma in primary-care settings in Alberta.

## Results

### Inter-rater reliability of the two expert physicians

Inter-rater reliability was established from an initial review of 100 random records provided by CPCSSN. Before any discussion took place, the level of agreement between their judgements about caseness was 97%, and the Kappa (inter-rater agreement) score was 0.88 (*P*⩽0.01). In circumstances where the two reviewers were uncertain or disagreed about the diagnosis, a physician from the study team (A.J.C.) reviewed the record, and the three physicians discussed the case until an agreement was reached. This served to standardise judgement before the 1000 record review.

### Validation metrics

Table 1 summarises the validation metrics for sensitivity, specificity, positive predictive value (PPV) and negative predictive value (NPV).

## Discussion

### Main findings

The results of this study indicate that the sensitivity, specificity, PPV and NPV of the algorithm are strong overall. Impressive specificity was found across all age groups, indicating that the algorithm finds few false-positive cases. The values remain relatively consistent by age group, indicating that the algorithm is adequate at estimating the true prevalence of asthma in both younger and older children.

### Interpretation of findings in relation to previously published work

Case-finding algorithms have previously been validated by CPCSSN for a number of chronic conditions, including COPD.^4,5^ These studies utilised a similar process whereby original patient charts were audited by primary-care physicians to determine whether patients had any of the CPCSSN indexed conditions and then compared with a CPCSSN case definition diagnosis. However, ours is the first study to use CPCSSN records rather than original EMR charts to validate a disease diagnosis.

In Canada, the majority of studies validating a diagnosis of childhood asthma have focused on identifying patients with asthma using administrative and prescription data^6,7^ as well as parental questionnaires.^8^ Our algorithm yielded higher specificity and lower sensitivity when compared with a case validation for children with asthma that utilised a single diagnosis code from primary-care administrative data in Ontario, Canada (sensitivity of 91.4% and specificity of 82.9%).^7^ In a more comparable study, Xi *et al*.^9^ tested the accuracy of an EMR-based search algorithm to identify patients over age 16 years with asthma and found a sensitivity and specificity of 90.2% and 83.9%, respectively, using their best search strategy. Our algorithm performed better in identifying patients up to 17 years of age.

Previous research in Canada has also demonstrated the effectiveness of using algorithm-defined cases to identify patients with other chronic conditions using data from EMRs. Krysko *et al*.^10^ found an EMR-based algorithm to identify multiple sclerosis performed well (91.5% sensitivity and 100% specificity) and could be used as an accurate tool in primary-care settings. Ivers and colleagues found that an EMR-based algorithm accurately identified patients with Ischaemic Heart Disease (72.4% sensitivity and 99.3% specificity) while outperforming other methods of identification.^11^ In addition, Widdifield *et al*.^12^ determined their algorithm for identifying patients with rheumatoid arthritis to be accurate and applicable for use in primary care (74.4% sensitivity, 99.9% specificity). Overall, this previous evidence further demonstrates the potential and feasibility of using EMR-based algorithms to identify patients in primary-care practices.

### Strengths and limitations of this study

This study is the first to validate childhood asthma in primary care using CPCSSN records, and has been proven to be successful in validating both cases and noncases. A major strength of the study was the utilisation of SAPCReN-CPCSSN records that allowed the study access to a large sample of patients of all ages within the southern Alberta primary-care population. CPCSSN practices also agree to quarterly medical audits, which allow for easy and regular access to data for disease diagnosis and surveillance. Further advantages to this approach include the use of cleaned data, anonymity for all patients, and both time and cost efficient access to data. However, the use of the CPCSSN database may also limit the generalisability of the findings, as a recent study determined that CPCSSN data were ‘only somewhat representative of the general Canadian population,’^13^ although representativeness was higher with respect to the population under age 19 years. Furthermore, the applicability of the algorithm-defined case definition may be limited to primary-care practices that are members of CPCSSN, as the algorithms are not designed for use with data from the uncleaned EMR records of primary-care practices outside of the network.

In addition to the limitations associated with the use of CPCSSN data, the study was limited by the variables and quality of data extracted from patient charts. As CPCSSN applies data cleaning and transformation algorithms to their data sets, the physician reviewers were unable to access full, original EMR charts and corresponding notes that may have better supported their diagnosis. The study was also limited to using variables extracted by CPCSSN. For example, although referral text is included in the data elements, referral documents are not. Finally, the quality of the data collected was dependent on the data as recorded in the family physicians’ offices. This limitation applies to the data used for the case definition algorithms as well as data abstracted from the EMR records. The study team had no control over how or what was recorded in the office or EMR, and no attempt to contact the patients to evaluate the quality of the data externally was made. As such, asthma may have been misclassified and under-diagnosed by both the physician reviewers and case-finding algorithm due to incomplete data or poor documentation.

### Implications for future research, policy and practice

This study provides an accurate definition of asthma and identification of children with asthma in primary care. Using this validated case definition, children with asthma can be identified by clinicians and researchers for improved practice/group care. Conversely, those not meeting the definition may be re-assessed by their physician and removed from unnecessary treatment. This validated definition will lead to quality improvement opportunities and further research and policy implications relating to asthma management in primary care. For instance, the findings from this study will be used in preparation of a randomised control trial of an asthma management pathway for children in Alberta aimed at improving asthma care throughout the province.^14^ Furthermore, the validation of an accurate asthma case-finding algorithm has value in providing a more accurate picture into the primary-care prevalence, diagnosis and management of asthma in Alberta and Canada.

## Conclusions

In summary, the case definition and algorithm for paediatric asthma presented in this study is valid for research purposes in CPCSSN primary-care practices. The validation of the diagnosis in the SAPCReN-CPCSSN database will be a foundation for primary-care asthma research in the province and in the country as a whole.

## Materials and methods

### Study sample

The CPCSSN is a national system of primary-care research networks that utilise EMR data in chronic disease surveillance.^3^ It currently consists of 10 primary-care research networks (PCRNs), including the Southern Alberta Primary Care Research Network (SAPCReN).^15^ Consent is sought from participating family physicians to allow the extraction of patient data by their local network. Extracted data are subjected to automated cleaning and standardising algorithms in order to ensure consistency in content and format through time and irrespective of data entry practices or source EMRs. Furthermore, CPCSSN data are considered anonymised as no directly identifiable patient information is extracted from the patient’s EMR and de-identification algorithms are applied to the data set.^3^ CPCSSN securely collects and combines fully de-identified data shared from the primary-care practice-based research networks and stores the data in a secure, central data repository at Queen’s University (ON, Canada).^3^ The present study obtained approval from the Health Research Ethics Board (HREB) at the University of Alberta (Edmonton, AB, Canada).

The population in this study included the random, anonymised records of 1000 paediatric patients (aged 1–17 years) of any gender and all health status, who were registered in SAPCReN-CPCSSN at the time of data extraction. The sample was randomly selected from patient records that belonged to consenting providers and met the inclusion criteria for the study. Assuming worst-case sensitivity and specificity estimates of 50%, the sample of 1000 patient records ensured an overall precision value better than 10% given a prevalence of asthma of around 9% for adults and 13% for children as suggested by Statistics Canada.^16^

Infants under age 1 year were excluded from the study by the researchers because of the issues associated with diagnosis in this age group and the multiplicity of other causes of respiratory symptoms.^1^ The Canadian Thoracic Society and the Canadian Paediatric Society state that asthma can be satisfactorily diagnosed in children from 1 to 5 years of age.^2^ Lung function testing, bronchial challenge and other physiologic tests used to confirm asthma diagnoses in older children are not possible under age 6 years and are not consistently available for use in primary care.^1,2^ It is also clinically difficult to distinguish asthma from other common conditions in children under 1 year of age. For instance, it may be difficult to differentiate symptoms of asthma from bronchiolitis, which shares similar signs of airflow obstruction, and from viral respiratory illness.^2^ Other alternative causes of respiratory symptoms in infants under 1 year of age could include recurrent upper respiratory tract infections (URTIs) with postnasal drip, croup, pertussis or gastroesophageal reflux disease.^2^

### Record review process

To establish a ‘gold standard’ of disease definition, two experienced primary-care physicians (EA and SF) were recruited. Along with two physician researchers (AJC and SMRK-B), they developed a data evaluation sheet (Supplementary Appendix 1) to use in determining ‘caseness’ using current literature and clinical experience. The two physicians were blinded to the algorithmic diagnosis. They were then provided with the same sample of 100 records that were reviewed separately and used to determine the degree of inter-rater reliability and allow for any variances to be discussed. Following this, they were each provided with a sample of 500 records (1000 total) from the SAPCReN-CPCSSN database (data up to 30 June 2015).

The physicians independently examined each record to identify the diagnosis of asthma by considering and weighing criteria including patient age, gender, diagnostic labels, the use of medications, diagnostic tests and referrals. Diagnostic label criteria included the documentation of ‘asthma’ or an International Classification of Diseases, Ninth Revision (ICD-9) diagnostic code (493 and derivatives) in the encounter text, problem list, or billing entry. Concurrent diagnostic labels (e.g. acute bronchitis) over time were also considered. The medication criteria included one or more prescriptions for an inhaled corticosteroid, long-acting β-agonist, short-acting β-agonist, corticosteroid pill, combined inhaled corticosteroid and long-acting bronchodilator, or leukotriene receptor antagonist. Criteria further included referrals to a respiratory medicine service or allergist, as well as requests for a respiratory test for the diagnosis of asthma (e.g. spirometry).

After reviewing their records, each physician completed an excel spreadsheet that included CPCSSN ID, case (yes or no) and criteria supporting their decision. The physicians agreed that there was a category of ‘suspected asthma’ records, which were likely to indicate caseness but could not be definitively diagnosed as such from the available data. A few of these records were from very young patients (1–2 years old) and had very limited data in their record; others lacked an adequate number of indicators to suggest or support a diagnosis. After discussion between the expert physicians and a third physician from the study team (AJC), it was determined that cases identified as ‘suspected asthma’ indicated a high likelihood of an asthma diagnosis for all clinical purposes and should thus be considered to have the disease for comparison with the algorithm-defined cases.

### Case definition

A case definition was created by a study physician (AJC) as well as a registered nurse experienced in asthma education (HS) and a second physician (SMRK-B)—both external to the study—using current guidelines^2^ and variables existing in the SAPCReN-CPCSSN records. This process involved three drafts, with the three developers meeting each time to reach consensus. The final case definition used a combination of ICD-9 codes and textual variables drawn from various sections of the EMR, including billing, encounter diagnosis, health conditions and prescribed medications. The definition included any occurrence of ‘asthma’, although excluding any occurrence of ‘asthma query’. Only persons between the ages of 1 and 17 years inclusive were eligible for inclusion. Furthermore, classification of childhood asthma required more than a single prescribed medicine, or a single prescribed medicine along with at least one other criterion (billing, encounter diagnosis or health condition). As such, although we acknowledge that children who did not present to the physician would not have been included in the sample, we attempted to include symptomatic children without an asthma diagnosis by including those who had asthma medications prescribed despite having no diagnostic label in their record. Table 2 presents the operational case definition for childhood asthma.

To develop the algorithm, a researcher blinded to the physician review (CD) used the previously determined case definition and the Case Finder feature of version 4.1 of the Data Presentation Tool (DPT), a remote access interface to a SAPCReN-CPCSSN database. The DPT was implemented with FileMaker Pro software, version 14 (FileMaker, Santa Clara, CA, USA) and loaded with the SAPCReN-CPCSSN 2015-Q2 data for the same 1000 patients reviewed by the expert physicians. The case-defined algorithm was then refined several times using an iterative process, until no new search or exclusion criteria were found necessary. For instance, the exclusion of ‘asthma query’ required several revisions in order to capture all variability as recorded in the records.

### Comparing algorithm results with record review

The case definition was validated by comparing the algorithm results against the gold standard physician record review. Results from the physician record review (case or noncase) were tabulated and inserted alongside those of the algorithm in a 2×2 table for each age category. Although the case definition includes only children aged 1–17 years, the algorithm was also run for ages between 3–17 and 6–17 years as these age groups are clinically important and may have different management characteristics. The corresponding sensitivity, specificity and positive and negative predictive values (PPVs and NPVs) were then calculated.

## Funding

This study was supported by an unrestricted grant from Astra Zeneca Canada. The study funder was not involved in study design; in the collection, analysis, and interpretation of data; in the writing of the report; or in the decision to submit the paper for publication.

## Figures and Tables

**Table 1:** Summary of the validation metrics for ages 1–17, 3–17 and 6–17 years

*Age range*	*Total, n*	*Results*				*Sensitivity, % (95% CI)*	*Specificity, % (95% CI)*	*Predictive value, % (95% CI)*	
		*TP*	*FP*	*TN*	*FN*			+	−
1–17 years	1000	125	12	845	18	87.4 (80.6–92.2)	98.6 (97.5–99.2)	91.2 (84.9–95.2)	97.9 (96.7–98.7)
3–17 years	904	124	11	752	17	87.9 (81.1–92.6)	98.6 (97.4–99.2)	91.9 (85.6–95.7)	97.8 (96.4–98.7)
6–17 years	720	108	11	585	16	87.1 (79.6–92.2)	98.2 (96.6–99.0)	90.8 (83.7–95.1)	97.3 (95.6–98.4)

Abbreviations: CI, confidence interval; FN, false negative; FP, false positive; *n*, number; TN, true negative; TP, true positive.

**Table 2:** Operational case definition for paediatric asthma

*Billing diagnosis*	*Encounter diagnosis*	*Health conditions*	*Medications*	
Any occurrence of the following ICD-9 code:	Any occurrence of the following ICD-9 code:	Any occurrence of the following ICD-9 code:	At least two prescriptions of the following:	
493—asthma	493—asthma	493—asthma	*Drug name*	*ATC code*
	Any occurrence of the following text: asth*	Any occurrence of the following text: asth*	Beclomethasone	R03BA01
			Budesonide	R03BA02
			Fluticasone	R03BA05
	The following are excluded: *asthma*query* *query*asthma* *asthma*?* *?*asthma*	The following are excluded: *asthma*query* *query*asthma* *asthma*?* *?*asthma*	Triamcinolone	R03BA06
			Mometasone	R03BA07
			Ciclesonide	R03BA08
			Salmeterol and fluticasone	R03AK06
			Formoterol and budesonide	R03AK07
				
			Formoterol and beclometasone	R03AK08
			Formoterol and mometasone	R03AK09
			Salbutamol	R03AC02
			Terbutaline	R03AC03
			Fenoterol	R03AC04
			Salmeterol	R03AC12
			Formoterol	R03AC13
			Indacaterol	R03AC18
			Zafirlukast	R03DC01
			Montelukast	R03DC03
			Dexamethasone	H02AB02
			Prednisolone	H02AB06
			Prednisone	H02AB07
			Ipratropium bromide	R03BB01
