# Supplementary material for: Development of a validated algorithm for the diagnosis of paediatric asthma in electronic medical records
Source: NPJ Prim Care Respir Med. 2016 Nov 24;26:16085–. doi: 10.1038/npjpcrm.2016.85 (PMC5122312; doi:10.1038/npjpcrm.2016.85)
Supplement: Supplemental Information [file npjpcrm201685-s1.doc]

**Asthma Algorithm Validation Study
Data Evaluation Sheet**

**Chart number:**

Date of Birth:_________________________; Age:_____________

Gender: Male
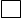
 Female

**Does this patient have an asthma diagnosis** (based on next pages, items I-V):


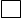
 Yes
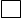
 No
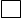
 Maybe

If *yes/maybe*, continue to fill out the evaluation, if *no* go to the next chart.

**Asthma investigation approach (please mark):**

**I:** **Actual diagnosis of asthma is documented in the chart:**

ICD code

Final diagnosis list

Billing code

**II:** **One or more of these drugs have been prescribed for the patient (at least one time):**

1. Inhaled corticosteroid
   - Beclomethasone dipropionate HFA (QVAR)
   - Budesonide (Pumicort Turbuhaler)
   - Ciclesonide (Alvesco)
   - Fluticasone (Flovent MDI and spacer; Flovent Diskus)
   - Mometasone (Asmanex Twisthaler)
   - Triamcinolate acetonide
2. Long acting beta agonists
   - salmeterol (Serevent)
   - formoterol (Foradil, Oxeze)
3. Short acting beta agonist
   - salbutamol (Ventolin, Apo-Salvent, Novo Salmol, Gen-salbutamol, Alti-Salbutamol, Airomir)
   - fenoterol hydrobromide (Berotec)
   - terbutaline sulfate (Bricanyl® inhaler)
4. Corticosteroid pills
   - Prednisone, Prednisolone (PediaPred)
   - Dexamethasone (Decadron)
5. Combined inhaled corticosteroids and long-acting bronchodilators

- Symbicort: Made of a corticosteroid (budesonide / Pulmicort) plus a long-acting bronchodilator (formoterol / Oxese)
- Advair - (fluticasone/salmerterol)

1. leukotriene receptor antagonists
   - zafirlukast (Accolate),
   - montelukast (Singulair)
2. Theophylline
   - TheoDur
   - Uniphyll
   - Phyllocontin
   - TheoLair
3. Other drugs in the above groups (1-7) that are not mentioned specify the name and the last time taken.

____________________________________________________________

**III) Respiratory test requested for diagnosis of asthma**

- Spirometry (FEV1/FVC)
- Peak expiratory flow variability
- Positive challenge test

Notes: ________________________________________________________________________________________________________________________________________________

**IV)** **Hospital admission because of asthma attack**

Notes (including emergency department attendance):

________________________________________________________________________________________________________________________________________________

**V) Referrals to pediatric pulmonologist/allergologist/respiratory unit**

________________________________________________________________________________________________________________________________________________

**Important notes in symptoms and history of the patients:**

**Asthma triggers**

- Exercise
- Cold air
- Smog
- pollution and fumes
- Hot, humid air
- Scents
- Emotional upsets, anxiety
- Smoking
- Pets
- Dust
- Viral respiratory tract infections
- Drug: please specify:___________________________________

Notes: ________________________________________________________________________________________________________________________________________________

**Allergy**

Specify: ___________________________________

**Related symptoms/ signs**

- Shortness of breath
- Wheeze
- Cough
- Chest pain/ tightness

**Asthma related conditions**

- COPD
- GERD
- Depression /Anxiety disorders
- Obesity: specify BMI at diagnosis if applicable: ______________________

**Family history:**

- Please specify: ___________________________________

**Night time symptoms:**

- Please specify: ___________________________________

**Smoking:** YES ______NO_________

Any evidence of passive smoker; specify: _____________________________

**Cardiovascular condition:**

Congenital heart disease; specify__________________

**Other major diseases (e.g., cancer, autoimmune, connective tissue):**

Specify: ______________________________________________________

**Date of first diagnosis (asthma):**_________________________

**Is there any note regarding asthma recovery (please explain):**

________________________________________________________________________________________________________________________________________________

**Other important notes in the chart:**

________________________________________________________________________________________________________________________________________________
